# NG2/CSPG4 regulates cartilage degeneration during TMJ osteoarthritis

**DOI:** 10.3389/fdmed.2022.1004942

**Published:** 2022-10-25

**Authors:** David A. Reed, Yan Zhao, Mina Bagheri Varzaneh, Jun Soo Shin, Jacob Rozynek, Michael Miloro, Michael Han

**Affiliations:** 1 Department of Oral Biology, University of Illinois Chicago, Chicago, IL, United States,; 2 Department of Oral and Maxillofacial Surgery, University of Illinois Chicago, Chicago, IL, United States

**Keywords:** temporomandibuar joint, mechanotranduction, cartilage, cell-matrix interactions, NG2/CSPG4, ERK signaling, chondrocyte, arthopathy

## Abstract

Changes in the mechanical homeostasis of the temporomandibular joint (TMJ) can lead to the initiation and progression of degenerative arthropathies such as osteoarthritis (OA). Cells sense and engage with their mechanical microenvironment through interactions with the extracellular matrix. In the mandibular condylar cartilage, the pericellular microenvironment is composed of type VI collagen. NG2/CSPG4 is a transmembrane proteoglycan that binds with type VI collagen, and has been implicated in the cell stress response through mechanical loading-sensitive signaling networks including ERK 1/2. The objective of this study is to define the role of NG2/CSPG4 in the initiation and progression of TMJ OA and to determine if NG2/CSPG4 engages ERK 1/2 in a mechanical loading dependent manner. In vivo, we induced TMJ OA in control and NG2/CSPG4 knockout mice using a surgical destabilization approach. In control mice, NG2/CSPG4 is depleted during the early stages of TMJ OA and NG2/CSPG4 knockout mice have more severe cartilage degeneration, elevated expression of key OA proteases, and suppression of OA matrix synthesis genes. In vitro, we characterized the transcriptome and protein from control and NG2/CSPG4 knockout cells and found significant dysregulation of the ERK 1/2 signaling axis. To characterize the mechanobiological response of NG2/CSPG4, we applied mechanical loads on cell-agarose-collagen scaffolds using a compression bioreactor and illustrate that NG2/CSPG4 knockout cells fail to mechanically activate ERK 1/2 and are associated with changes in the expression of the same key OA biomarkers measured *in vivo*. Together, these findings implicate NG2/CSPG4 in the mechanical homeostasis of TMJ cartilage and in the progression of degenerative arthropathies including OA.

## Introduction

Osteoarthritis (OA) is the most common pathology affecting the temporomandibular joint (TMJ) and is characterized by extracellular matrix and cartilage degradation that is associated with limited joint mobility, pain, and diminished quality of life (1). While the etiopathogenesis of TMJ OA has yet to be fully resolved, there is consensus that a change in mechanical homeostasis is a strong initiating condition of cartilage degeneration. Mechanical forces on a tissue are transduced to the cell through the extracellular matrix (2). The pericellular matrix is a division of the extra cellular matrix that encompasses the cell. In mandibular cartilage, the pericellular matrix is composed of collagen IV, laminin, perlecan, and collagen VI (3). Collagen VI is a micro- fibrillar collagen that regulates the material properties of the pericellular matrix (4). During TMJ OA, there are changes in the distribution, organization, and quantity of collagen VI, indicating that the mechanical microenvironment of the cell may be affected during the progression of TMJ OA (5–7).

Neuron-Glial antigen 2 (AN2 in mice; Chondroitin sulfate proteoglycan 4 in humans, NG2/CSPG4) is an N-linked type I transmembrane glycoprotein with chondroitin sulfate proteoglycan components on the ectodomain. NG2/CSPG4 binds with collagen VI (8, 9). The full-length core protein without chondroitin sulfate chains is represented by a 300 kDa band. Ectodomain shedding occurs through proteolytic processing by MMP13 (10), MMP14 (11), MMP9 (12), and/or ADAM10 (13), generating a truncated, cell-associated/membrane-tethered fragment between 260 and 275 kDa (11, 13, 14). MMP13 is significantly elevated after mechanical loading on mandibular fibrochondrocytes (15, 16), suggesting that NG2/CSPG4 ectodomain shedding is contextually linked with changes in mechanical homeostasis. NG2/CSPG4 ectodomain shedding is believed to initiate the release of the intercellular domain through an α-secretase mediated process of regulated intramembrane proteolysis (17). This extracellular surface shedding and intracellular processing potentiates both inside-out and outside- in signaling (18, 19).

In mandibular condylar cartilage, NG2/CSPG4 binds with pericellular type VI collagen in articular/superficial layer cells. During the progression of TMJ OA, NG2/CSPG4 translocates from the cell membrane to the cytosol (20). In other cell types, NG2/CSPG4 internalization occurs at focal adhesions and is associated with cell-matrix dynamics and motility (19, 21). The mechanism and role of NG2/CSPG4 internalization and proteolytic processing in cartilage homeostasis has not been mechanistically resolved. However, NG2/CSPG4 has been implicated in collagen binding in limb OA chondrocytes (22) and chondrosarcomas (23). Together, these data indicate that NG2/CSPG4 signaling may be important for regulating the health and disease of cartilage in the TMJ and elsewhere.

NG2/CSPG4 regulates a wide variety of cellular processes including migration, survival, apoptosis, and differentiation, due in part to multivalent nature of the inside-out and outside- in signaling modalities of the molecule (18, 19). The intracellular C-terminal domain has acceptor sites for the extracellular signal-regulated kinases (ERK) 1/2 and protein kinase C-alpha (PKC-α) (24), binding domains for multi-PZD domain protein 1 (MUPP-1) (25), interactions with integrins (9), and regulates mTORC1 (18, 19). NG2/CSPG4 mediated regulation of the ERK 1/2 signaling cascade is particularly important for studies on mechanotransduction. The ERK 1/2 signaling axis is a critical regulator of the mechanobiological response in chondrocytes (26–31). Impact injury to cartilage activates ERK 1/2 signaling axis through MAPK, promoting key OA proteases including MMP13 and ADAMTS5 (32). ERK 1/2 signaling is activated by the release of FGF2 from the pericellular matrix (33), a growth factor that binds to NG2/CSPG4 (34). ERK 1/2 is further implicated in the progression of OA by regulating the differentiation cascade of chondrocytes (35, 36), hypertrophy (37), osteoblast differentiation (38), and regulating the fate of mesenchymal stem cells in response to mechanical loading (39).

Together, these studies illustrate that NG2/CSPG4 may be a key mechanotransductive signaling hub in mandibular fibrochondrocytes during TMJ health and disease. NG2/CSPG4 influences ERK 1/2 signaling in a wide range of cells, and chondrocyte differentiation pathways in particular. ERK 1/2 can modulate key OA proteases in a mechanical loading dependent manner in mandibular and limb chondrocytes. Here we address a key gap in knowledge regarding if NG2/CSPG4 is a significant regulator of TMJ health and disease *in vivo* and if mechanical activation of ERK 1/2 signaling occurs in an NG2/CSPG4 dependent manner. We hypothesize that NG2/CSPG4 regulates cartilage degeneration during degenerative arthropathy and the mechanobiological activation ERK 1/2. Here we mechanistically interrogate the role of NG2/CSPG4 in the homeostasis of mandibular condylar cartilage using an *in vivo* preclinical surgical instability murine model of TMJ OA and an *in vitro* compression bioreactor mechanical loading model.

## Results

### TMJ OA is associated with loss of the NG2/CSGP4 ectodomain and an internalized intracellular domain

**FIGURE 1:**
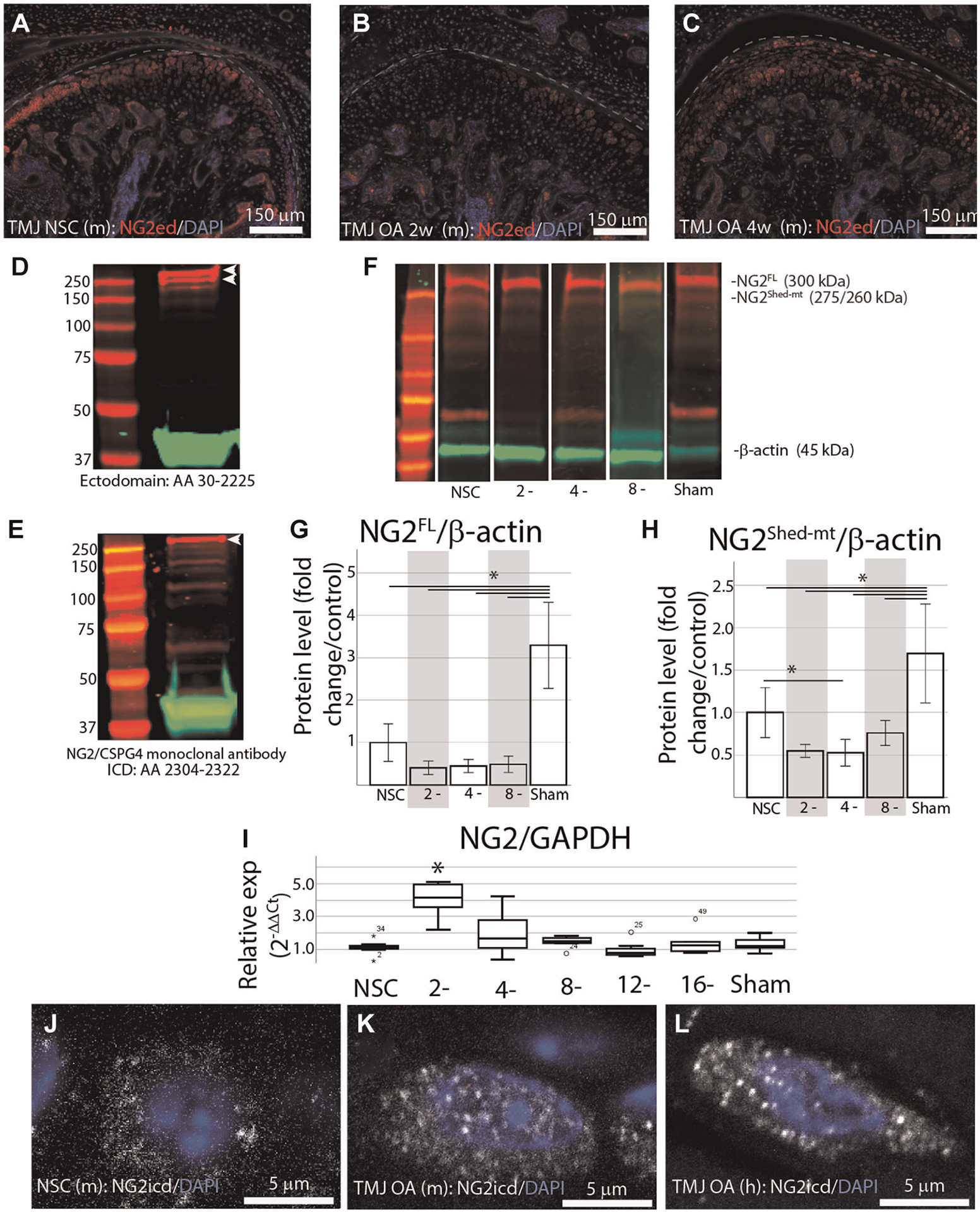
Early stage TMJ OA is associated with a loss of the NG2/CSPG4 ectodomain and internalization of the intercellular domain. (A) NG2/CSPG4 immunofluorescent staining from TMJ tissue in a non-surgical control mouse. (B) NG2/CSPG4 immunofluorescent staining from TMJ OA tissue 2-weeks after discectomy. (C) NG2/CSPG4 immunofluorescent staining from TMJ OA tissue 4-weeks after discectomy. (D) Western blot analysis from protein lysate extracted from primary mandibular fibrochondrocytes, separated on a 12% SDS PAGE gel, and immunolabeled using a polyclonal antibody raised against the NG2/CSPG4 ectodomain. (E) Western blot analysis from the same protein sample, run on the same gel, and immunolabeled using a monoclonal antibody raised against the NG2/CSPG4 intracellular domain. Note two distinct bands at 300–260 kDa with the polyclonal antibody and only one band representing the full-length protein on the monoclonal intracellular domain antibody. (F) Western blot from mouse tissue 2-, 4-, and 8-weeks after discectomy compared to non-surgical and sham controls immunolabeled with antibodies against NG2/CSPG4. (G,H) Quantification of the western blots from the full-length 300 kDa (G) and 260/275 kDa shed, membrane tethered (H) NG2/CSPG4 fragments. Note a significant reduction in the shed, membrane-tethered fragment a 4-weeks post-operative compared to the non-surgical control and a significant increase in full length and shed, membrane-tethered NG2 in the sham. *N* = 4/experimental group. (I) RT-qPCR on mRNA isolated from murine mandibular condylar cartilage illustrating that the expression level of NG2/CSPG4 is significantly elevated 2-weeks post-operative but is not significantly different from the non-surgical control through the rest of the progression of TMJ OA. *N* = 6/experimental group. (J) The spatial distribution of the NG2/CSPG4 intracellular domain (NG2icd) in an articular/superficial layer mandibular fibrochondrocyte from non-surgical control TMJ tissue. (K) The spatial distribution of NG2icd in articular/superficial layer mandibular fibrochondrocytes 8-weeks after discectomy. (L) The spatial distribution of the NG2icd in an articular/superficial layer mandibular fibrochondrocyte from a human patient with TMJ OA. *p < 0.05.

To characterize how NG2/CSPG4 is altered in during the progression of TMJ OA *in vivo*, we analyzed mandibular condyles from wild type (c57 BL/6J) mice at 2- and 4-weeks after discectomy, and compared them with age matched non-surgical controls with immunofluorescence using an antibody against the NG2/CSPG4 ectodomain. These data illustrate that NG2/CSPG4 levels are decreased in the mandibular condylar cartilage following discectomy (Figures 1A–C). The NG2/CSGP4 ectodomain can be present as a 300 kDa full-length protein, or a 260/275 kDa shed, membrane tethered fragment that lacks the intracellular domain. To confirm that the 260/275 kDa fragment lacks the intracellular domain, we performed western blot analysis on protein samples from primary mandibular fibrochondrocytes immunolabeled using a polyclonal antibody labeled against the ectodomain and a monoclonal antibody labeled against the intracellular domain. These data illustrate that the monoclonal antibody only labeled the 300 kDa full-length fragment, confirming that the 260/275 kDa fragment lacks the intracellular domain (Figures 1D,E). To characterize NG2/CSPG4 during the progression of TMJ OA, we analyzed isolated mandibular condyles by western blot from 2-, 4-, and 8-week TMJ OA samples and compared them with non-surgical and sham controls. Western blot analysis illustrates that the sham controls have significantly more full length and shed, membrane-tethered NG2/CSPG4 fragments than the non-surgical control. In the TMJ OA samples, there is a non-significant reduction in full length NG2/CSPG4 at all stages and a significant reduction in the shed, membrane tethered fragment at 4-weeks after discectomy (Figures 1F–H). RT-qPCR illustrates that NG2/CSPG4 gene expression is significantly increased at 2-weeks after discectomy but returns to the level of the non-surgical control shortly after (Figure 1I). We had previously illustrated TMJ OA is associated with elevated levels of cytosolic NG2/CSPG4 in articular layer cells of the mandibular condylar cartilage using a polyclonal antibody raised against the full length protein (20). Using immunohistochemistry with an antibody specific to the NG2/CSPG4 intracellular domain, we illustrate that TMJ OA is associated with high levels of the NG2/CSPG4 intracellular domain in the cytosol in both a preclinical murine model (Figures 1J,K) and in a sample from a human TMJ OA patient undergoing total joint replacement (Figure 1L). Together, these data illustrate that *in vivo* TMJ OA is associated with loss of NG2/CSPG4 ectodomain fragments and an increase in the presence of the NG2/CSPG4 intracellular domain in the cytosol. These data also indicate that NG2/CSPG4 localized to the cytosol is characteristic of clinical TMJ dysfunction.

### NG2/CSPG4 knockout mice have higher levels of cartilage degeneration during early stage TMJ OA and have increased levels of key OA biomarkers

**FIGURE 2:**
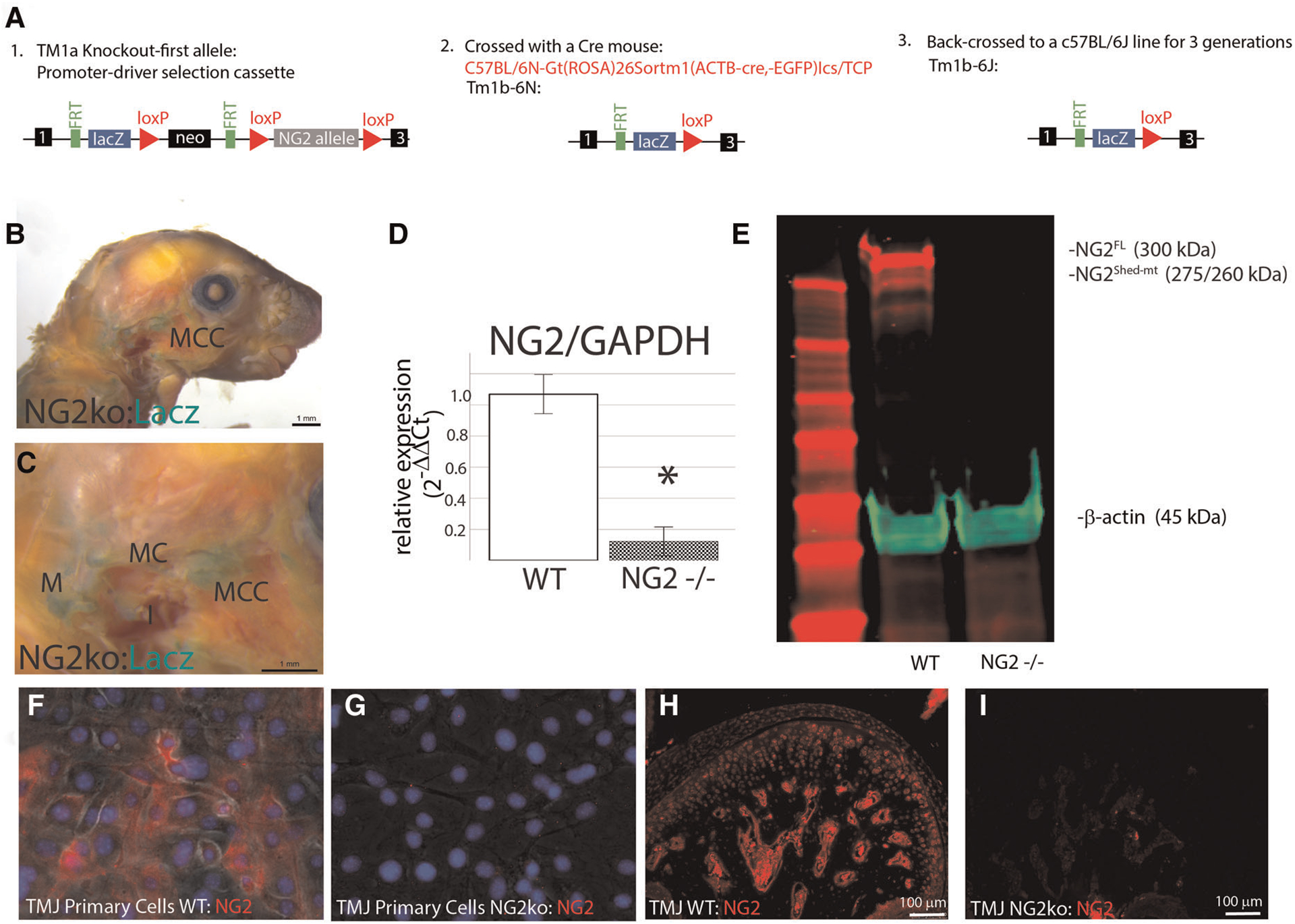
Validation of the NG2/CSPG4 knockout mice and primary cells. (A) Flow diagram illustrating the generation of the NG2/CSPG4 mouse line. The ES cell cryorecovery, genotyping, and Cre breeding was carried out by the KOMP repository. The TM1b-6N line was provided for the study. This line was backcrossed to a c57BL/6J line for 3 generations so that the data from the knockout mouse could be compared with control c57BL/6J mice. After three generations of backcrossing, a homozygous knockout-breeding colony of the Tm1b-6J was established. (B) Lac-Z reporter staining illustrating the activation of the transgenic construct in the TMJ. (C) Zoomed region of interest near the TMJ illustrating positive Lac-z staining in secondary cartilage like the mandibular condylar cartilage (MCC) and primary cartilages including Meckel’s cartilage (MC) and the malleus (M). (D) RT-qPRC from the TMJ of WT and NG2/CSPG4 knockout mice illustrating the loss of NG2/CSPG4 expression in the transgenic mice. Data were normalized to GAPDH using the *ΔΔ*Cq method. (E) Western blot analysis of the TMJ from WT c57 BL/6J and NG2/CSPG4 knockout mice illustrating the loss of both the 300 and 260/275 kDa fragments of NG2/CSPG4 in the knockout tissue. (F) Immunostaining of NG2/CSPG4 from primary cells from mandibular condylar cartilage of wild type c57 BL/6J mice. (G) Immunostaining of NG2/CSPG4 from primary cells from mandibular condylar cartilage of NG2/CSPG4 knockout mice illustrating loss of the protein. (H) Immunostaining of NG2/CSPG4 from the TMJ of WT c57 BL/6J mice. (I) Immunostaining of NG2/CSPG4 from the NG2/CSPG4 knockout mice illustrating loss of the protein in the LacZ positive tissues. *N* = 4/experimental group. *p < 0.05.

**FIGURE 3:**
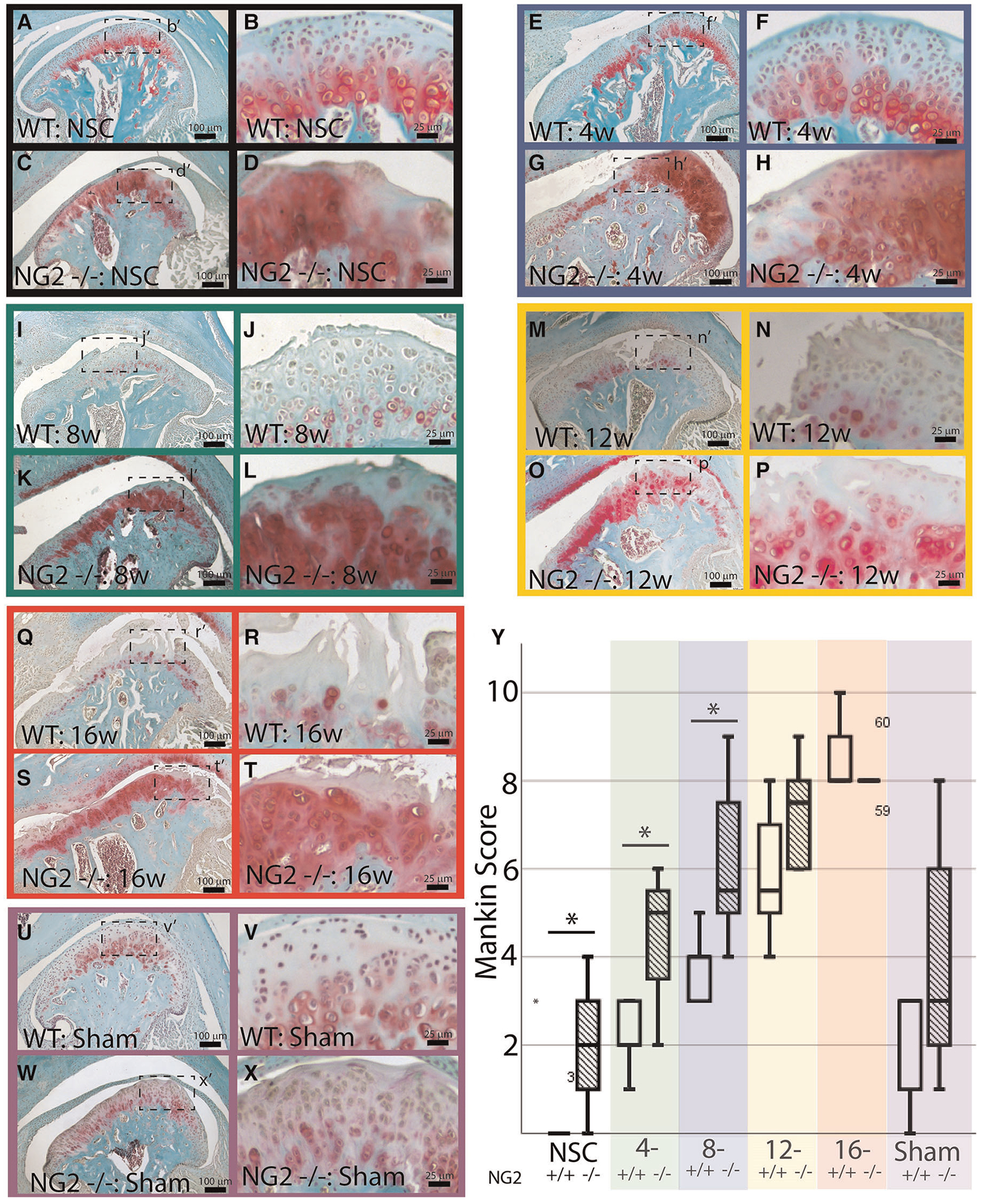
NG2/CSPG4 knockout tissues have higher rate of cartilage degeneration. Safranin-o/fast green staining of skeletally mature wild-type (WT) and NG2/CSPG4 knockout (NG2 −/−) TMJ tissues illustrating the progression of cartilage degeneration at several experimental time points. All images are from the surgical side of the animals (right joint) and are representative images from a sample size of four. Images were taken at two magnifications for each sample. Each experimental time point is color-coded. (A–D) non-surgical control mice (NSC) is in black. (E–H) 4-week post-discectomy (4w) is in blue. (I–L) 8-week post-discectomy (8w) is in green. (M–P) 12-week post-discectomy (12w) is in yellow. (Q–T) 16-week post-discectomy (16w) is in red. (U–X) 16-week sham control (sham) is in purple. (Y) Quantification of cartilage degeneration using a Modified Mankin Score comparing wild type and NG2/CSPG4 knockout TMJ’s at each stage of cartilage degeneration illustrating significantly increased degeneration scores in the NG2/CSPG4 knockout tissues in the early stages OA progression. The color code on the graph corresponds to the color coded experimental time points. *N* = 4/genotype and experimental group. *p < 0.05.

**FIGURE 4:**
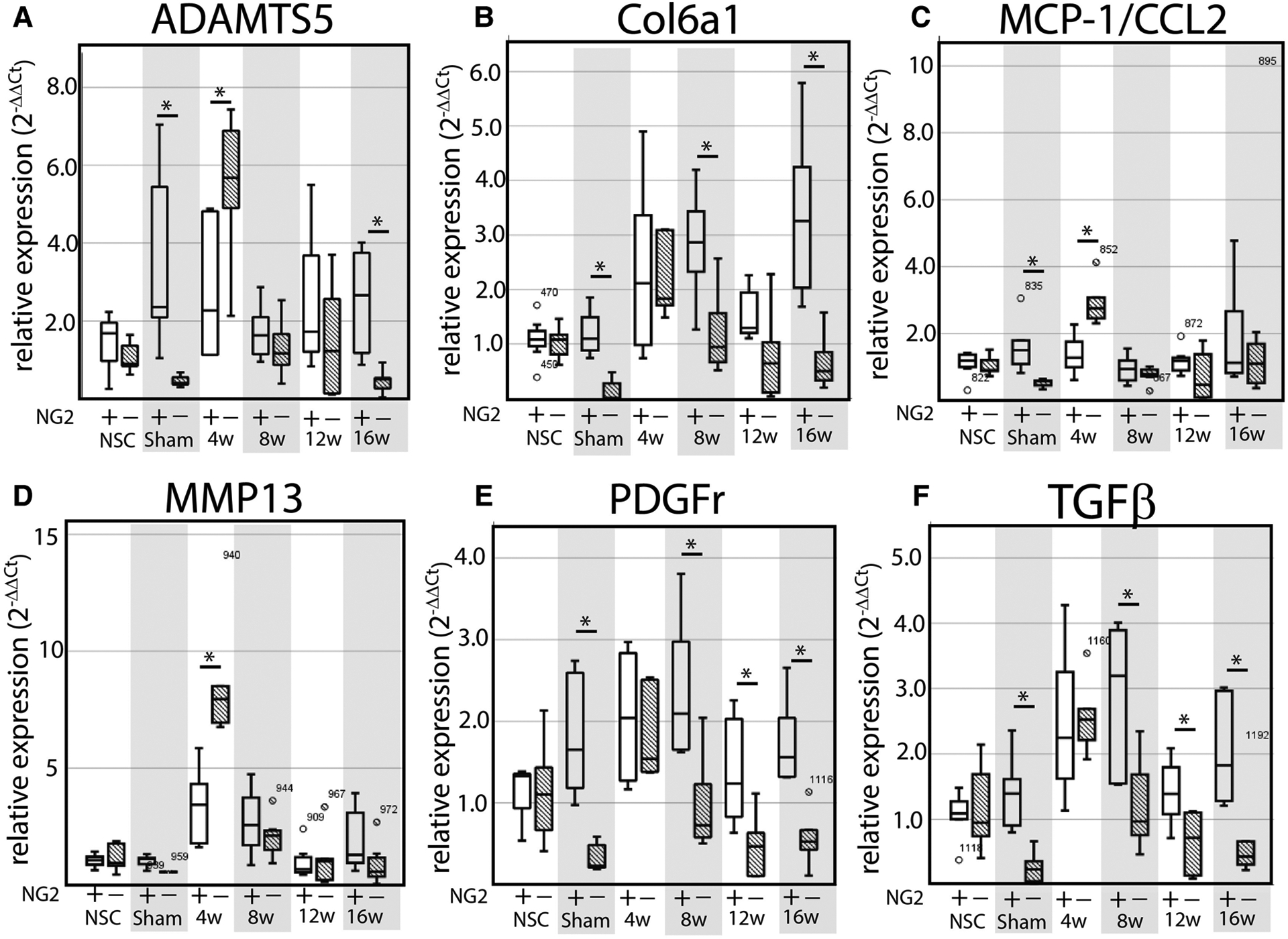
NG2/CSPG4 knockout tissues have elevated OA proteases and suppressed matrix synthesis gene expression during TMJ OA. (A–F) RT-qPCR from mRNA isolated from the mandibular condylar cartilage of wild type and NG2/CSPG4 knockout mice in non-surgical and sham controls and in 4-, 8-, 12-, and 16-week discectomy mice illustrating changes in the expression of ADAMTS5 (A), Col6a1 (B), MCP-1/CCL2 (C), MMP13 (D), NG2/CSPG4 (E), PDGFr (F), and TGFβ (G). Data are standardized to the NSC sample for each genetic background and normalized to GAPDH using the ΔΔCq method. *N* = 6/genotype and experimental group. *p < 0.05.

This study uses NG2/CSPG4 global knockout mice backcrossed to a c57 BL6/J line (Figure 2A). Global expression of NG2/CSPG4 was assessed by whole mount staining for a LacZ reporter inserted in a promoter driven cassette. This staining illustrates that NG2/CSPG4 is present in both primary and secondary cartilages in the craniofacial skeleton, including Meckel’s and mandibular condylar cartilage respectively (Figures 2B,C). NG2/CSGP4 knockout mice are viable through skeletal maturity. Loss of NG2/CSPG4 protein in LacZ positive tissues was confirmed in both TMJ tissue and primary cells at the gene, mRNA, and protein level (Figures 2D–I). To determine if NG2/CSPG4 regulates the rate of cartilage degeneration, TMJ OA was induced in wild-type control and NG2/CSPG4 knockout mice and the tissue was stained by safranin-o/fast green. Cartilage degeneration was graded using a modified Mankin Scoring matrix [see references (15, 16)]. NG2/CSPG4 knockout mice score significantly higher on degeneration scores than the wild-type controls for the non-surgical control mice and at 4- and 8-weeks after discectomy. This increase in the degeneration score is due to abnormal spatial distribution of proteoglycans, higher levels of hypocellularity in the articular layer cartilage, and a loss of cartilage integrity. There is no significant difference in the 12- and 16-week samples when there is high hypocellularity (Figures 3A–Y). To determine if NG2/CSPG4 knockout mice had an increase in key OA biomarkers compared to wild type controls, we screened control and TMJ OA mandibular condylar cartilage samples using RT-qPCR. In 16-week post-operative sham controls, NG2/CSPG4 knockout tissue was associated with a significant suppression of ADAMTS5, COL6a1, MCP1, PDGFrβ, and TGFβ. A moderate tissue response in the sham controls is expected in this mouse model since the joint cavity is opened and there is an inflammatory response of the tissue to the surgery (5, 16, 20). At 4-weeks after discectomy, the NG2/CSPG4 knockout tissue was associated with significant elevation of key OA proteases ADAMTS5, and MMP13 and the chemokine CCL2/MCP1. At 8- and 16-weeks after discectomy, the NG2/CSPG4 knockout tissue has significant suppression of matrix synthesis genes Col6a1, PDFGrβ, and TGFβ (Figure 4). Together, these data implicate NG2/CSPG4 as a key regulator of cartilage homeostasis during TMJ health and disease.

### Constrained static compression promotes the loss of the NG2/CSPG4 ectodomain and clathrin-mediated internalization of the intracellular domain

**FIGURE 5:**
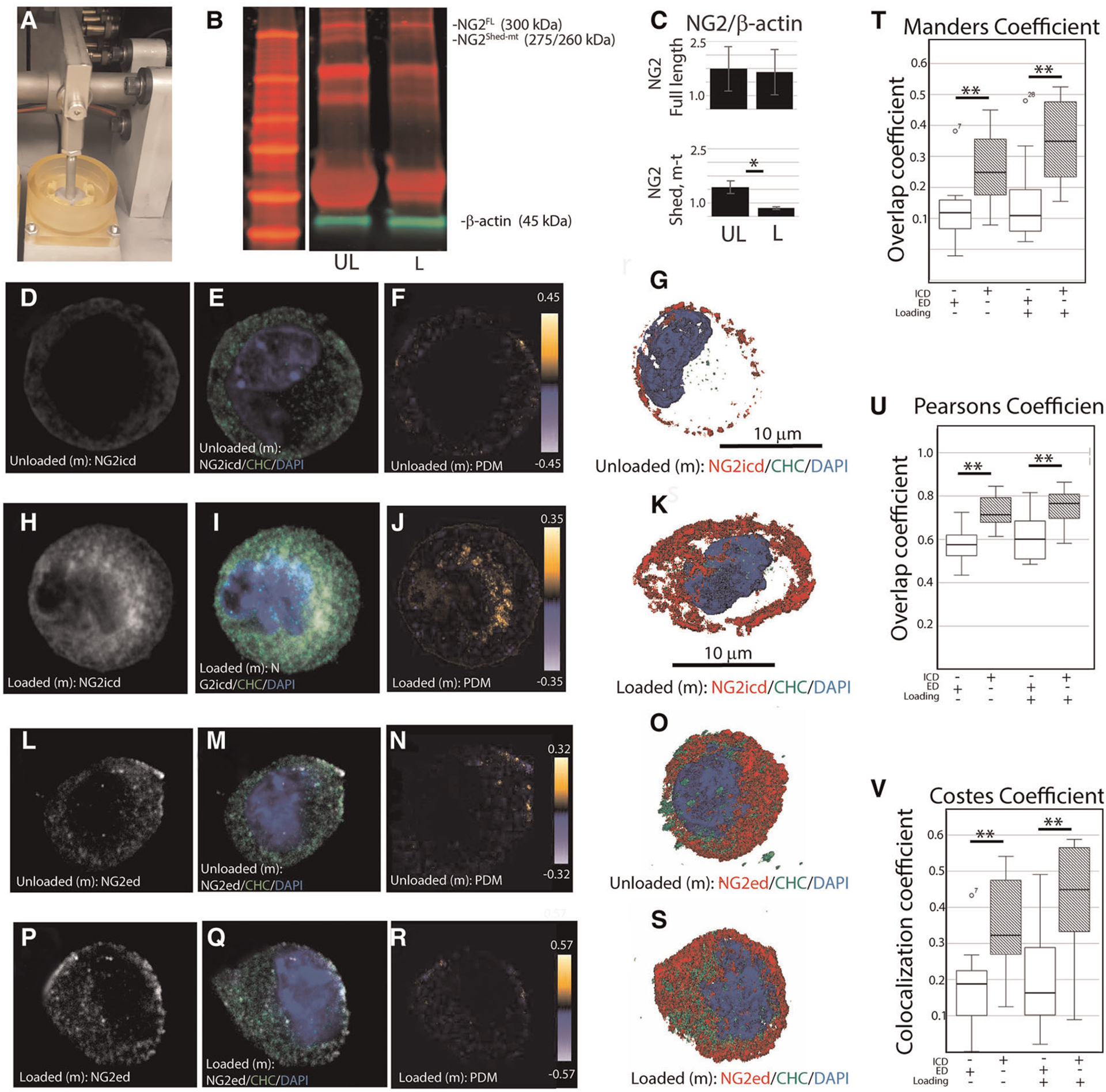
Mechanical loading promotes loss of the NG2/CSPG4 ectodomain and clathrin mediated internalization of the intracellular domain. (A) A compression bioreactor used to apply constrained static compression in mandibular fibrochondrocytes *in vitro*. (B) Western blot analysis of unloaded (UL) and loaded (L) wild type primary mandibular fibrochondrocytes illustrating loss of the 260/275 kDa shed, membrane-tethered NG2/CSPG4 fragment after compression. (C) Quantification of NG2/CSPG4 protein illustrating a significant reduction in the 260/275 kDa fragment after compression. *N* = 4/experimental group. *p < 0.05. (D) The spatial distribution of the NG2/CSPG4 intracellular domain (NG2icd) in an unloaded mandibular fibrochondrocyte. (E) The spatial distribution of clathrin heavy chain in an unloaded mandibular fibrochondrocyte. (F) The overlap of NG2icd/CHC in an unloaded mandibular fibrochondrocyte using the product of the difference from the mean illustrating overlap near the cell membrane. (G) Z-stack three-dimensional reconstruction of NG2icd/CHC/DAPI in an unloaded mandibular fibrochondrocyte. (H) The spatial distribution of the NG2icd in a mandibular fibrochondrocyte loaded under constrained compression. (I) The spatial distribution of CHC in a mandibular fibrochondrocyte loaded under constrained compression. (J) The overlap of NG2icd/CHC in a mandibular fibrochondrocyte loaded under constrained static compression using the product of the difference from the mean illustrating colocalization inside of the cell. (K) Z-stack three-dimensional reconstruction of NG2icd/CHC/DAPI in a mandibular fibrochondrocyte loaded under constrained static compression. (L) The spatial distribution of the NG2/CSPG4 ectodomain (NG2ed) in an unloaded mandibular fibrochondrocyte. (M) The spatial distribution of clathrin heavy chain (CHC) in an unloaded mandibular fibrochondrocyte. (N) The overlap of NG2ed/CHC in an unloaded mandibular fibrochondrocyte using the product of the difference from the mean illustrating limited overlap near the cell membrane. (O) Z-stack three-dimensional reconstruction of NG2ed/CHC/DAPI in an unloaded mandibular fibrochondrocyte. (P) The spatial distribution of the NG2ed in a mandibular fibrochondrocyte loaded under constrained compression. (Q) The spatial distribution of CHC in a mandibular fibrochondrocyte loaded under constrained compression. (R) The overlap of NG2ed/CHC in a mandibular fibrochondrocyte loaded under constrained static compression using the product of the difference from the mean illustrating limited overlap near the cell membrane. (S) Z-stack three-dimensional reconstruction of NG2ed/CHC/DAPI in a mandibular fibrochondrocyte loaded under constrained static compression. (T) Mander’s overlap coefficient between NG2/CSPG4 and CHC using the NG2icd and NG2ed antibody in loaded and unloaded cells. (U) Pearson’s overlap coefficient between NG2/CSPG4 and CHC using the NG2icd and NG2ed antibodies. (V) Costes Colocalization coefficient between NG2/CSPG4 and CHC using the NG2icd and NG2ed antibodies. Overlap and colocalization between values between NG2icd/CHC and NG2ed/CHC are significant different using all methods. *N* = 3/experimental group. **p < 0.001. All Costes colocalization values are significant when compared to random background noise at 25 iterations. *N* = 5/experimental group; *p* > 0.95.

To evaluate if NG2/CSPG4 internalization can be induced through mechanical stress, primary mandibular fibrochondrocytes were embedded in a three-dimensional agarose-collagen scaffold, loaded in a compression bioreactor, and evaluated by western blot and immunohistochemistry (Figure 5A). Western blot analysis of NG2/CSPG4 illustrates that unloaded cells contain a full-length 300 kDa fragment and a 260/275 kDa fragment indicating separation from the intracellular domain and retention of the shed, membrane tethered ectodomain (11, 13). After mechanical loading, there are significantly lower levels of the 260/275 kDa band and retention of the full-length protein (Figures 5B,C), matching the pattern measured during the early stages of TMJ OA *in vivo*. To determine if mechanical loading is associated with an increase in intracellular NG2/CSPG4, we conducted confocal immunostaining on unloaded and loaded primary mandibular fibrochondrocytes using both the intracellular and ectodomain antibodies against NG2/CSPG4. The distribution of these antigens within the cell were visualized using a three-dimensional z-stack (Figures 5D–S). To quantify if these NG2/CSPG4 fragments were being actively transported into the cell, we calculated colocalization coefficients of NG2/CSPG4 with a marker for endocytosis, clathrin heavy chain (CHC), using three methods: the Mander’s overlap coefficient, the Pearson’s overlap coefficient, and the Costes Colocalization method. All three methods illustrate a high level of agreement that the NG2/CSPG4 intracellular domain and CHC significantly colocalize inside of the cell following mechanical loading, with all observed Costes colocalization values significant compared to randomized noise (*N* = 5; *P* > 0.95). Conversely, there is less support for colocalization between the NG2/CSPG4 ectodomain and CHC, with lower colocalization values than the intracellular domain in all cases (Figures 5T–V). These findings illustrate that the NG2/CSPG4 ectodomain is altered in response to mechanical loading, losing the shed, membrane-tethered fragment and increasing the amount of cytosolic NG2/CSPG4 intracellular domain through clathrin mediated endocytic pathways.

### NG2/CSPG4 knockout cells are transcriptionally distinct from control cells

**FIGURE 6:**
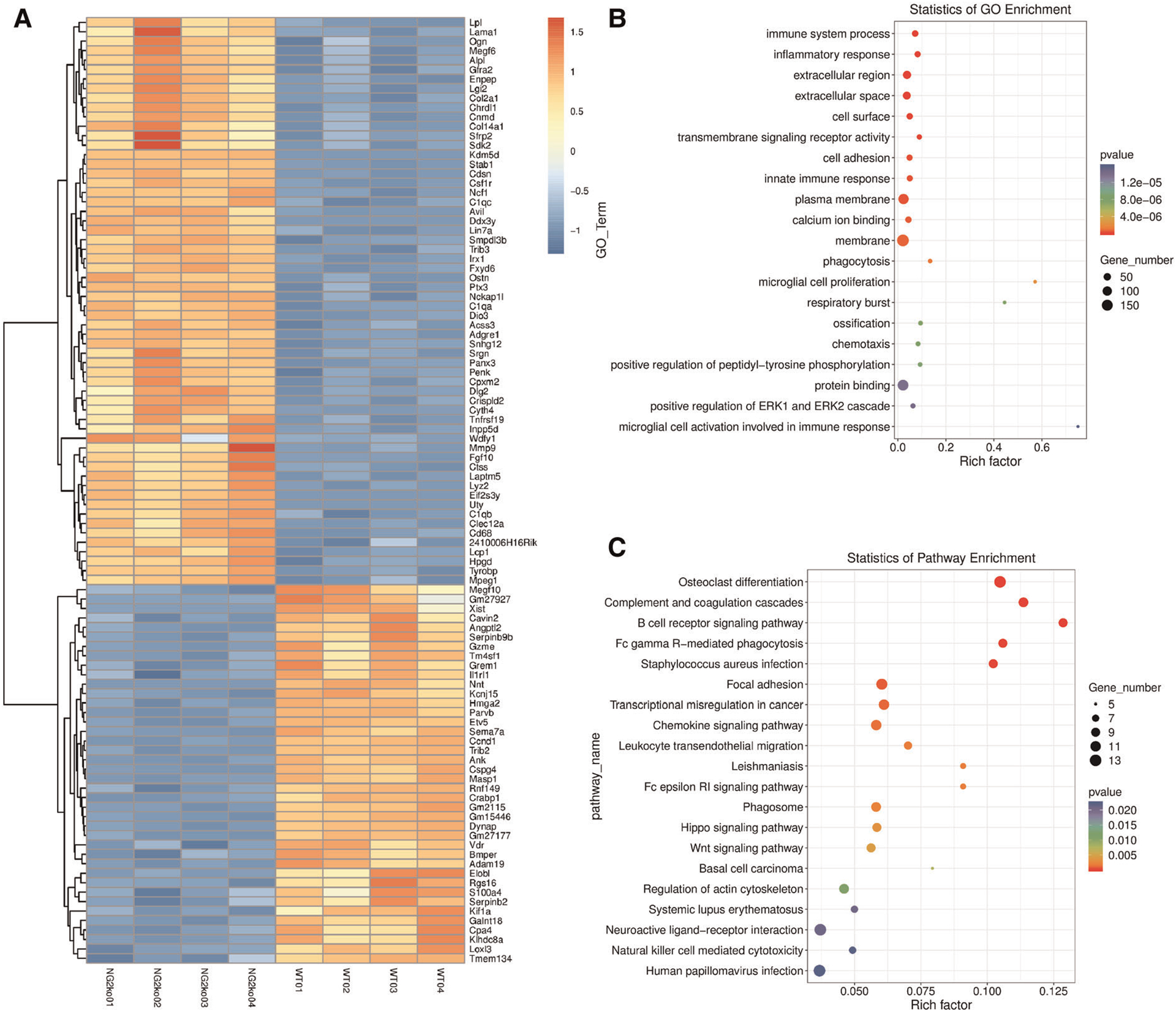
NG2/CSPG4 knockout mandibular fibrochondrocytes have a unique transcriptional profile. (A) Heat map of differentially expressed genes and transcripts in wild type (WT) and NG2/CSPG4 knockout (NG2ko) cells from a total RNA seq analysis. (B) A gene ontology enrichment analysis from the total RNA-seq data illustrating significant upregulation of the gene ontology network “positive regulation of ERK1 and ERK2 cascade” as well as a number of other cell adhesion and protein binding families. (C) A KEGG pathway enrichment analysis from the total RNA-seq data. *N* = 4/genotype group.

To screen for transcriptional changes related to the loss of NG2/CSPG4, isolated mRNA was evaluated by total RNAseq. Differential gene expression was characterized by a heat map and yielded robust and significant differences in genes expression with the five most upregulated genes being Eif2s3y, Uty, Ddx3y, Kdm5d, and Clec12a. Other notable upregulated genes include MMP9, Col14a1, Fgf10, Alp1, and Col2a1 (Figure 6A). Twenty significant gene ontology enrichment groups were identified including a number of enriched pathways related to the extracellular region/space and “positive regulation of the ERK1 and ERK2 cascade”. This gene ontology enrichment group can indicate that ERK 1/2 either is suppressed or enriched (Figure 6B). Further analysis of the expression data using a KEGG enrichment analysis identified engagement with the focal adhesion pathway, the hippo pathway, and the Wnt pathway (Figure 6C). GO enrichment analysis identifying significant changes in the ERK 1/2 pathway is of note since ERK 1/2 is a mechanical loading-sensitive signaling network implicated in the progression of OA and chondrocyte differentiation.

### NG2/CSPG4 knockout cells are associated with suppressed of p44/p42 MAPK (ERK 1/2)

**FIGURE 7:**
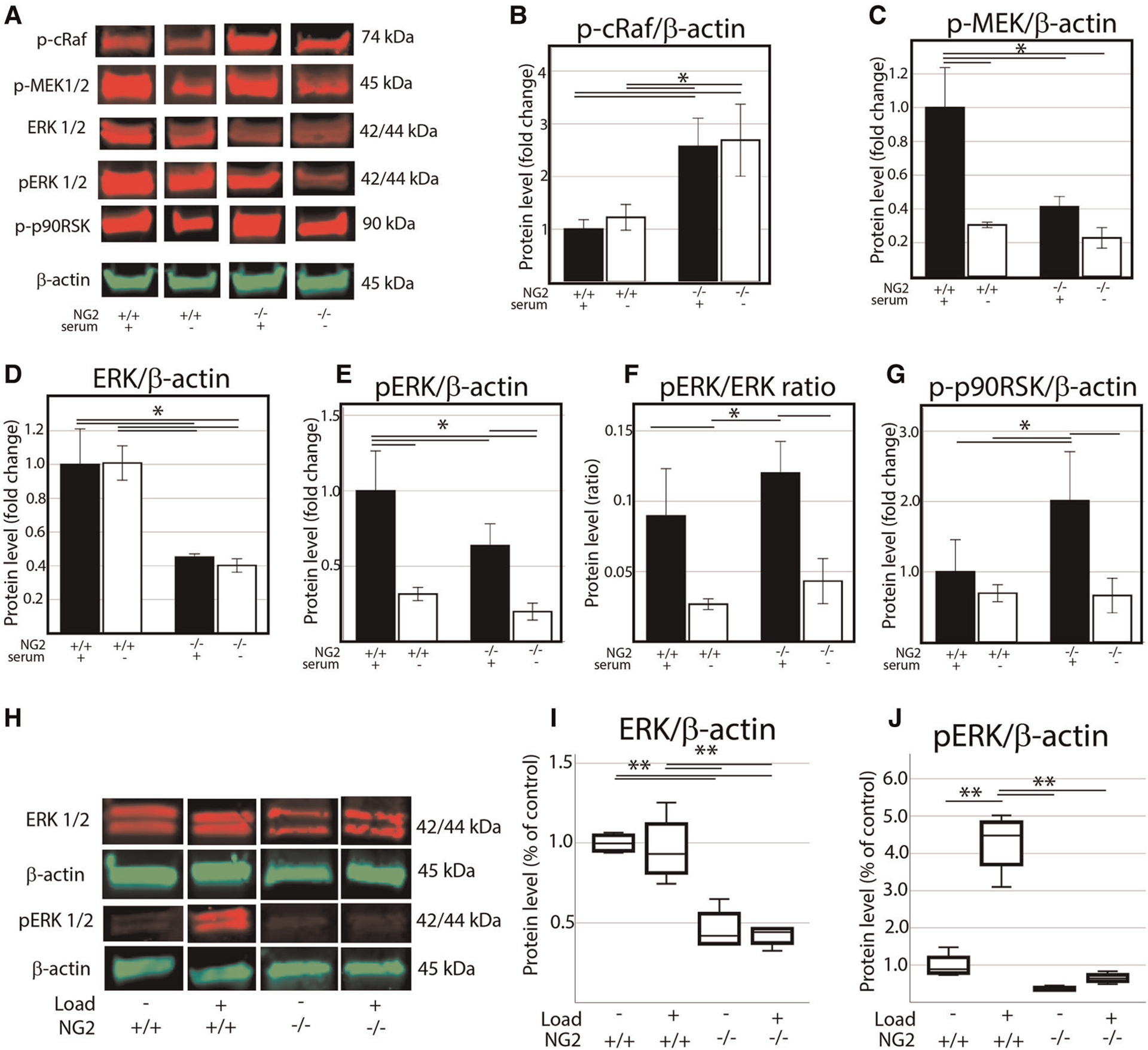
NG2/CSPG4 knockout mandibular fibrochondrocytes have suppressed p44/p42 MAPK (ERK 1/2) signaling and deficiencies in the mechanical activation of ERK 1/2. (A) Western blot analysis of the ERK 1/2 signaling axis in wild type and NG2/CSPG4 knockout (NG2ko) primary cells cultured in normal and low serum conditions. (B) Quantification of the phosphorylated cRaf western blot. (C) Quantification of the phosphorylated MEK 1/2 western blot. (D) Quantification of the total ERK 1/2 western blot. (E) Quantification of the phosphorylated ERK 1/2 western blot. (F) Quantification of the ratio of phosphorylated ERK/total ERK. (G) Quantification of the phosphorylated p90RSK western blot. (H) Western blot analysis of unloaded and loaded wild type and NG2/CSPG4 knockout primary mandibular illustrating protein levels of ERK 1/2 and phosphorylated ERK 1/2 (I–J) Quantification of western blot analysis illustrating that NG2ko cells have significantly lower levels of total ERK 1/2 and fail to phosphorylate ERK 1/2 in response to constrained static compressive loading. *N* = 4/genotype and experimental group. *p < 0.05; **p < 0.01.

To further characterize how ERK 1/2 signaling was altered in the NG2/CSPG4 knockout mice, we screened protein samples from wild-type and NG2/CSPG4 knockout primary cells by western blot for upstream regulators of the ERK 1/2 pathway including cRaf and MEK 1/2, for ERK 1/2, and the downstream target p90RSK. Cells were cultured in both normal media and serum starvation conditions. NG2/CSPG4 knockout increased phosphorylation of cRaf in a serum independent manner. Phosphorylation of MEK 1/2 was suppressed in an NG2/CSPG4 and serum dependent manner. ERK 1/2 and phosphorylated ERK 1/2 were both suppressed in an NG2/CSPG4 dependent manner but were not impacted by the presence of serum. Phosphorylation of p90RSK, a downstream target of ERK 1/2, was elevated in an NG2/CSPG4 dependent manner only in the presence of serum (Figures 7A–G). Together, these data illustrate that knocking out NG2/CSPG4 in mandibular fibrochondrocytes alters the transcriptional profile of the cell and impacts the regulation of ERK 1/2 signaling, consistent with findings in other cell types (18, 24). Since ERK 1/2 is a key mechanotransductive molecule and since the NG2/CSPG4 intracellular domain contains an ERK 1/2 acceptor site, we performed western blot analysis of ERK 1/2 and phosphorylated ERK 1/2 in loaded and unloaded wild type and NG2/CSPG4 knockout cells. These data illustrate that mechanical loading activates ERK 1/2 signaling in mandibular fibrochondrocytes and that NG2/CSPG4 knockout cells have a significant reduction total ERK 1/2 and in phosphorylated ERK 1/2 (Figures 7H–J).

### Mechanical loading promotes the expression of key OA biomarkers in an NG2/CSPG4 and ERK 1/2 dependent manner

**FIGURE 8:**
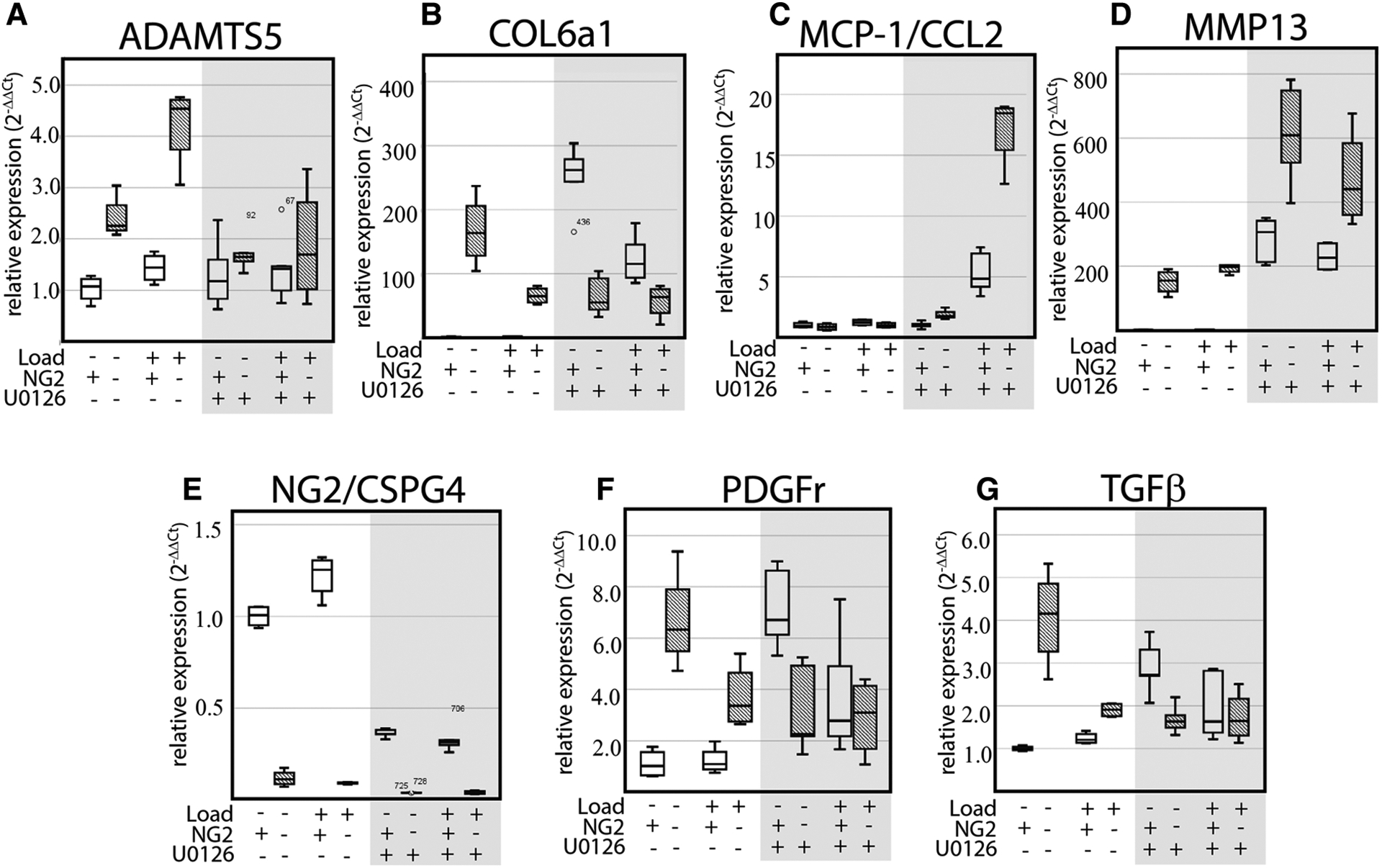
Mechanical loading alters the expression of key OA biomarkers in an NG2 and ERK 1/2 dependent manner. (A–G) RT-qPCR of WT and NG2ko cells treated in a compression bioreactor with and without the ERK 1/2 small molecule inhibitor U0126 illustrating NG2/CSPG4, loading, and ERK 1/2 dependent changes in a key OA biomarkers including ADAMTS5 (A), Col6a1 (B), MCP-1/CCL2 (C), MMP13 (D), NG2/CSPG4 (E), PDGFr (F), and TGFβ (G). Data standardized to the unloaded wild type sample and normalized to GAPDH using the ΔΔCq method. *N* = 4/genotype and experimental group. Significance values from multi-group comparisons are reported in the Supplementary Materials.

To evaluate if mechanical loading can alter the expression of key OA and matrix synthesis genes in an ERK 1/2 and/or NG2/CSPG4 dependent manner, wild type and NG2/CSPG4 knockout primary cells were seeded in a collagen-agarose scaffold, loaded in the compression bioreactor with or without the ERK 1/2 inhibitor U0126, and evaluated by RT-qPCR. NG2/CSPG4 knockout resulted in an increase in the expression of the OA biomarkers ADAMTS5 and MMP13, with ADAMTS5 responding in a mechanical loading dependent manner. Further, NG2/CSPG4 knockout resulted in an overall increase in matrix synthesis markers Col6a1, PDGFr, and TGFβ compared to the wild type controls and mechanical loading dependent suppression. When ERK 1/2 was inhibited using U0126, a small molecule functionally antagonizing AP-1 transcriptional activity, ADAMTS5 expression was rescued, while MMP13 expression of the wild type controls was elevated to the levels of the NG2/CSPG4 knockout cells. Similarly, inhibiting ERK 1/2 with U0126 elevated the expression of matrix synthesis biomarkers Col6a1, PDGFr, and TGFβ in wild type controls to levels matching the NG2/CSPG4 knockout cells. The U0126 treatment significantly elevated the expression of the chemokine MCP1/CCL2 in NG2/CSPG4 knockout cells in a mechanical loading dependent manner (Figure 8; *N* = 4; *p* < 0.05; significance provided in Supplementary Materials). Taken together, these data illustrate that NG2/CSPG4 knockout cells and the inhibition of ERK increases the expression of key OA proteases and suppresses the expression of key matrix synthesis genes.

## Discussion

Here we illustrate that NG2/CSPG4 has an important role for the initiation and progression of TMJ OA, displaying subcellular dynamics in response to mechanical loading and engaging the mechanical loading-sensitive signaling network, ERK 1/2. Our data illustrate, using both *in vivo* and *in vitro* approaches, that changes in mechanical homeostasis and OA are associated with the loss of the shed, membrane-tethered NG2/CSPG4 fragment and the internalization of the NG2/CSPG4 intracellular domain. Using transgenic knockout mice, we illustrate that the mechanical activation of the ERK 1/2 pathway is dependent on NG2/CSPG4 and that NG2/CSPG4 is a critical regulator of TMJ OA cartilage degeneration and pathophysiology. We also demonstrate that key OA proteases and matrix synthesis genes are dependent on NG2/CSPG4 and ERK 1/2 signaling.

Loss of NG2/CSPG4 ectodomain fragments is contextually linked with high levels of MMP13 expression in 4-week post-discectomy mice. The presence of this protease in the pericellular microenvironment may promote shedding and enzymatic processing of the NG2/CSPG4 ectodomain (15, 16). The concomitant increase in NG2/CSPG4 gene expression is likely a transient compensatory mechanism by the cell to replenish the loss of the extracellular protein following shedding, potentially through promoter activation by cytokines and hypoxic stress mediated transcription factors Sp1, Egr1, and Pax3 (18). The sham controls have significantly higher levels of both the full length and the shed, membrane-tethered fragment of NG2/CSPG4. In the sham control, the joint capsule is accessed surgically generating a moderate inflammatory/injury response from the tissue. We hypothesize that the increase in both NG2/CSGP4 fragments in the sham controls reflects transient inflammatory mediated promoter activation leading to the accumulation of membrane associated NG2/CSPG4 in the absence of proteases. This elevated inflammatory response in the sham controls may also explain the slightly elevated levels of cartilage degeneration, with Mankin Scores elevated due primarily to an increase in cell clustering and a slight reduction in proteoglycan (16, 20).

TMJ OA and mechanical loading is also associated with clathrin-mediated internalization of the NG2/CSPG4 intracellular domain. This subcellular pattern matches the distribution observed in diseased tissue *in vivo* both preclinically and clinically. The clathrin association with the NG2/CSPG4 intracellular domain, and not the ectodomain, supports the hypothesis that NG2/CSPG4 is released from the cell membrane through regulated intramembrane proteolysis. This regulatory process involves enzymatic processing of the ectodomain, followed by γ-secretase mediated release of the intracellular domain (17). In monolayer, shedding of the NG2/CSPG4 ectodomain is disassociated from a pericellular microenvironment. Our data were collected from a three-dimensional cell culture system, where the shed NG2/CSPG4 ectodomain is restricted to a pericellular microenvironment that has an enriched proteolytic milieu. In this microenvironment, enzymatic cleavage and processing of the shed, membrane-tethered ectodomain could generate even smaller fragments. An alternative explanation of the results is that the intracellular domain is being internalized along with some or all of the ectodomain through receptor mediate endocytic processing. Colocalization of the NG2/CSPG4 ectodomain with CHC was significantly lower than the intracellular domain, but not absent. There was limited overlap near the cell membrane. Post-translational changes and endocytic processing of NG2/CSPG4 could be occurring through multiple distinct processes. One important variable for mechanistically defining these processes is to characterize the presence and function of ectodomain associated chondroitin sulfate chains at the time of injury. Chondroitin sulfate chains have a potentially important biological role in NG2/CSPG4 functionality, promoting fragment retention near the membrane and/or serving as a mechanism for modulating the cell surface microdomain. NG2/CSPG4 could directly modulate cell surface microdomain mediated signaling due to the proteoglycans high affinity for FGF2 and PDGFaa, two growth factors implicated in OA pathophysiology (34, 40, 41).

Internalization and/or activation of the NG2/CSPG4 intracellular domain can further regulate key pathways implicated in OA progression through a PDZ binding motif (25), mTORC1 activation (17), and/or ERK 1/2 activation (24). This study focuses on NG2/CSPG4 engagement with ERK 1/2 through a putative D domain–docking site that acts as a substrate for ERK 1/2-mediated phosphorylation (24). In mandibular fibrochondrocytes, we demonstrate that NG2/CSPG4 knockout cells are associated with lower levels of total ERK 1/2 independent of mitogen activation through serum. Upstream of ERK 1/2, cRaf phosphorylation is elevated while MEK 1/2 phosphorylation is suppressed. Downstream of ERK 1/2, p90RSK phosphorylation was elevated in NG2/CSPG4 knockout cells. Interestingly, p90RSK regulates mTOR signaling, consistent with other reports linking NG2/CSPG4 with cell cycle kinetics through mTORC1 (17). Additional studies are needed to determine the role of ERK 1/2 and MEK 1/2 feedback loops and/or the coregulatory influence of protein kinase C signaling (24).

Activation of the ERK 1/2 signaling pathway can be achieved through multiple overlapping mechanisms including growth factors, cytokines, integrin engagement, and/or oxidative stress. Here we evaluated the role NG2/CSPG4 in the mechanical activation of ERK 1/2. Following static compression, NG2/CSPG4 knockout cells had suppressed total and phosphorylated ERK 1/2. NG2/CSPG4 knockout cells were also associated with mechanical loading dependent increases in key OA proteases and suppression of matrix synthesis genes when compared to loading in the wild type controls. Small molecule inhibition of ERK 1/2 through U0126 in wild-type cells resulted in elevated levels of matrix synthesis genes, matching the levels expressed in NG2/CSPG4 knockout cells. U0126 treatment of wild-type cells also resulted in a 60% reduction in the expression of NG2/CSPG4. U0126 treatment in NG2/CSPG4 knockout cells suppressed the aggrecanase ADAMTS5 and elevated the chemokine MCP1/CCL2 and collagenase MMP13. A similar pattern is found for protein levels of MCP-1 and MMP13, but not ADAMTS5. We hypothesize that this finding is related to U0126 functionally antagonizing the AP-1 transcription factor, upstream of MEK 1/2, which has a multifaceted role in regulating cell behavior. Taken together, these findings demonstrate that NG2/CSPG4 displays a complex and multifaceted intracellular processing mechanism resulting from the generation of fragments that potentiate variant-specific functions, including inside-out and outside-in signal transduction of mechanical loading in mandibular condylar cartilage.

The ERK 1/2 signaling pathway is a critical and well defined regulator of mechanical loading in all cell types, controlling proliferation, differentiation, and cell survival (42). In cartilage, ERK 1/2 inhibits early chondrocyte differentiation and hypertrophy, regulates osteo-chondroprogenitor cells and osteoclast formation (36), and is implicated in the progression of osteoarthritis (35, 43). ERK 1/2 activation during cartilage injury is hypothesized to be related to the release of growth factors from the pericellular matrix (33, 34) and mechanical engagement with the substrate (44). Our data are the first, to our knowledge, to implicate NG2/CSPG4 in the mechanobiological ERK 1/2 response, providing a mechanistic link from the extracellular microenvironment, through cell surface receptors, to transcription factors that promote discrete cell fate decisions affecting cartilage homeostasis. The regulation of ERK 1/2 following injury is a dynamic and complex network response. In limb cartilage impact models, ERK 1/2 phosphorylation was not detected 3 h after injury but was significantly elevated 24 h after injury (32). During the injury response of stromal cells, there is a strong initial wave of ERK 1/2 activation, cessation, then a second wave of ERK 1/2 activation that is maintained until wound closure (42). It is unknown if similar ERK 1/2 pulsations occur in the injury response of mandibular cartilage and if quantitative information modulating cell fate decisions are transduced to the cell through variations in the amplitude, frequency, and duration of the response. The complex temporal separation between NG2/CSPG4 expression, NG2/CSPG4 ectodomain shedding, and ERK 1/2 activation may obscure the signal-response relationship because of the multimodal nature of the initial ERK 1/2 signaling dynamics, including signal delay, amplitude, frequency, or duration.

While this study has focused on the TMJ, there is limited, but supporting, evidence that NG2/CSPG4 has an important role in arthropathies in primary cartilages such as the knee and hip. NG2/CSPG4 is present in the pre-cartilaginous mesenchyme of the developing rat limb, with expression decreasing in post-natal development (45). In post-natal tissues, NG2/CSPG4 is strongly upregulated at ossification fronts during both endochondral and intramembranous ossification (46). NG2/CSPG4 is also present in human adult osteoarthritic articular cartilage (47), impacting the ability of these cells to adhere with type VI collagen (22). Together, these studies support the hypothesis that NG2/CSPG4 functionality is conserved in both primary and secondary cartilages.

This study demonstrates that NG2/CSPG4 is an important regulator of cartilage health and disease in the TMJ, influencing mechanical loading-sensitive pathways such as ERK 1/2, and regulating the progression of cartilage degeneration during OA. These findings underscore that NG2/CSPG4 is a novel and underappreciated potential therapeutic target for degenerative arthropathies in the TMJ, potentially serving as an extracellular target of the ERK 1/2 signaling pathway. In cancer, NG2/CSPG4 has been proposed as an oncotarget for monoclonal antibodies (48) and for theranostics (49). Future studies will focus on how NG2/CSPG4 regulates other MAP kinases including MEK1/2, JNK, and p38 and evaluate NG2/CSPG4-ERK 1/2 signaling in a manner that characterizes changes in the amplitude, frequency, or duration of both molecules after following injury.

## Material and methods

### Control and NG2/CSPG4 knockout mice

Control mice from a C57 BL/6J background were purchased from Jackson Laboratory. Knockout mice were acquired from the KOMP repository [Cspg4tm1a(KOMP)Wtsi/Bcm] and were generated using the Knockout-first allele: Promoter driven section kit. The knockout mice used in this study were generated by cross breeding with a Cre expressing line to generate a reporter-tagged deletion allele. Mice heterogeneous for the reporter-tagged deletion allele were backcrossed to a C57 BL/6J line and then mated to generate a homozygous reporter-tagged deletion allele for NG2/CSPG4 (Figure 2A). All animals were housed together to minimize confounding conditions. To confirm LacZ reporter expression in target tissues, whole mount histochemical detection of β-galactosidase was performed in 10-day-old heads for control and NG2/CSPG4 knockout mice. NG2/CSPG4 knockout mice were viable through skeletal maturity with no strong developmental phenotype aside from those reported including increased lean body mass (Mousephenotype.org). A mild but significant phenotype in the TMJ cartilage is reported here. The use of all animal tissues followed an approved animal use protocol (UIC ACC #20-068).

### Primary mandibular fibrochondrocyte isolation

The isolation of primary mandibular fibrochondrocytes followed published methods (15, 20) using a protocol for isolating primary chondrocytes (50, 51). In short, mandibular condyles from 10 to 14 day old mice were isolated in CO2 independent collection medium (18045-088, Gibco, Gaithersburg, MD) supplemented with 25 mg/ml Plasmocin (ant-mmp, InVivoGen, San Diego, CA), 50 U/ml penicillin and 0.05 mg/ml streptomycin (P0781, Sigma, St. Louis, MO). Condyles were then washed in 1× sterile PBS and transferred to a digestion DMEM (11966-025, Gibco, Gaithersburg, MD) with 3 mg/ml type II collagenase (S004174, Worthington Biochemical, Lakewood, NJ) sterilized using a 0.2 μm filtered syringe. After 45-min, this digestion media was replaced with a 1.5 mg/ml collagenase solution and placed in an incubator at 5% CO_2_ at 37 °C for overnight digestion. After the digestion, cells were dispersed by gentle agitation, filtered through a 40 μm cell strainer, and centrifuged at 1000 g for 5 min at room temperature. The resulting pellet was re-suspended and then cultured in FBS supplemented advanced DMEM until confluent (12492-013, Gibco, Gaithersburg, MD). All experiments were carried within the first 6 passages of the cells. For all serum starvation experiments, cells were cultured in Opti-MEM reduced serum media for 24 h (31985, Gibco, Gaithersburg, MD).

### Generating cell-agarose-collagen scaffolds and mechanical loading

To define the role of mechanical loading on NG2/CSPG4 functionality, primary mandibular fibrochondrocytes were seeded in agarose-collagen scaffold and loaded in a compression bioreactor as previously described (15). In short, cell-agarose-collagen scaffolds were generated from a 5% low-gelling temperature agarose (A0701, Sigma, Burlington, MA) mixed in 5 ml 1× PBS. Once cooled to 45 °C, the 5% agarose solution was combined with 0.25 mg/ml rat tail collagen (A1048301, Thermo Fisher, Waltham, MA) and a cell suspension in 1 ml FBS supplemented DMEM to create a 4% agarose/collagen solution. A cell density of 4 × 10^5^/mm^3^ was used for all experiments (50). For casting, the cell-agarose/collagen solution was poured on a 1 mm glass spacer plate and covered to generate a uniform 1 mm thickness sheet (1653311, Bio-Rad, Des Plaines, IL). A punch was used to generate plugs measuring 17 mm in radius. The cell-agarose/collagen scaffold plugs cultured for 72 h in supplemented advanced DMEM (12492-013, Gibco, Gaithersburg, MD) at 37 °C and 5% CO_2_. For loading, all cell-agarose-collagen scaffolds were put in a compression bioreactor housed inside of a cell incubator and loaded in constrained, uniaxial compression at 2.5 N for 2 h. All loaded samples were compared to unloaded controls.

### Preclinical surgical instability model of TMJ OA

TMJ OA was induced by unilateral partial discectomy according to the methods in Xu et al. (16) and our previous publications (5, 15, 20, 52). In short, skeletally mature 16-week-old male and female mice from an NG2/CSPG4 knockout and control (c57 BL/6J) background were anesthetized with ketamine (100 mg/kg, Henry Schein, Dublin, Ohio) and xylazine (5 mg/kg, Akorn, Lake Forest, IL). The articular disc was excised on the right side of the animal. For excision of the articular disc, a 3–5 mm incision was made over TMJ, the lateral capsule was exposed, the articular disc excised using a posterior approach, and the joint was irrigated with sterile 1× PBS. Sham control surgeries were identical except the disc remained intact. For the sham control, the joint capsule is opened and a moderate inflammatory response is expected (16, 20). Tissues were collect a 4-, 8-, 12-, and 16-weeks after discectomy. Only joints on the surgical side of the animal were analyzed for this study. Only the right side was used for non-surgical control tissues. No randomization was used for this study. All *in vivo* data were compared with age matched non-surgical controls and 16-week sham controls. A sample size of 10 was used for each experimental time point, for a total of 120 control and NG2/CSPG4 knockout in the study. Sample size for each method of assessment was calculated using a sample size and power analysis. Each animal was considered an experimental unit. Experiments using vertebrate animals were approved by the University of Illinois at Chicago Animal Care Committee and performed in accordance with the relevant guidelines and regulations (UIC ACC #20-068). The study conforms to ARRIVE guidelines.

### Clinical human samples for comparison with preclinical data

Peri-articular tissue samples were collected intraoperatively from the clinical practice of the UIC Department of Oral and Maxillofacial Surgery during total TMJ replacement surgery. Tissue was collected from a confirmed case of advanced/end-stage TMJ OA where inflammatory or rheumatoid conditions were not the primary cause. All tissue was collected in the operating suite, placed in ice-cold 1× PBS, and prepared for histological analysis. All tissue collection was approved by the institutional review board of the University of Illinois at Chicago (IRB Protocol No. 2017-0033).

### Histomorphometry and immunohistological grading of cartilage degeneration

For histomorphometric grading of cartilage degeneration, tissue samples were fixed in 4% PFA overnight, decalcified with 4.5% EDTA for 28 days, paraffin embedded, and sectioned at 8 μm. Sections were stained using safranin-o/fast green and cartilage degeneration was staged using a Modified Mankin score according to published methods (15, 16, 20, 52). For immunohistochemistry, sections were deparaffinized, treated with 132.2 mM sodium borohydride, permeabilized with methanol and 0.5% Triton (v/v), blocked in 5% donkey serum (D9663, Sigma, St. Louis, MO) for 2 h, and incubated with primary antibodies against NG2/CSPG4 (1 : 200, AB5320, Sigma-Millipore, Santa Cruz, CA). All secondary labeling was with Alexa Fluor donkey anti-mouse 488 and donkey anti-rabbit 568 (1 : 500, Invitrogen, Invitrogen, Carlsbad, CA). Nuclei were label with DAPI (D9542-1MG, 1 μg/μl, Sigma, St. Louis, MO). Sections were imaged using an inverted fluorescent microscope using a 10× objective (DMI6000B, Leica, Buffalo Grove, IL). Laser intensity, gain, and magnification was standardized for all acquisitions. Brightness and contrast settings were standardized for all images during post-processing. All data were compared to a no primary antibody control and isotype control. Images were quantified by personnel blind to the study design, outcome assessment, and analysis. Control staining was carried out for the NG2/CSPG4 antibodies using NG2/CSGP4 knockout tissue (Figures 2F–G), staining without primary antibodies, and IgG controls [see reference (20)]. Autofluorescence was detectable in the subchondral vasculature but negligible in the mandibular condylar cartilage for the brightness and contrast settings used during data acquisition. Four biological replicates for each experimental group were used for the Modified Mankin scoring analysis. Modified Mankin Scoring method replicates previous published results (15, 16) and is provided in Supplementary Materials.

### Immunocytochemistry and colocalization analysis

Cell-agarose-collagen scaffolds were fixed overnight using Histochoice Tissue Fixative (VWRVH102, VWR, Radnor, PA), cryo-embedded using O.T.C. compound, and cryo-sectioned at 20 μm (CM1950, Leica, Buffalo Grove, IL). Immunostaining of the sample follows the methods described in the previous section using primary antibodies including a custom monoclonal antibody raised against the NG2/CSPG4 intracellular domain (1 : 200, GGQPDPELLQFCRTPNPALRNGQYWV, UIC Protein Core, Chicago, IL), and clathrin heavy chain (1 : 200, MA1-065, Thermo Fisher, Waltham, MA). All samples were imaged using a laser scanning confocal microscope with 63× oil immersion objective (LSM 710, Zeiss) using identical laser intensity, brightness, and gain standardized for all image acquisitions. Colocalization coefficients and fluorescence/channel intensity were calculated using ImageJ (53). Colocalization values using the Pearson’s overlap coefficient were compare (54). Colocalization was statistically validated using the Costes method. Four biological replicates for each experimental group.

### RT-qPCR

For quantifying gene expression changes from homogenized TMJ tissues, samples were placed in QIAzol (79306, Qiagen, Germantown, MD) and mRNA was isolated following manufacture protocol. To extract and purify mRNA from agarose samples, lysate was processed using the RNeasy Plant Mini Kit (74903, Qiagen, Germantown, MD). The plant kit was recommended for high integrity extraction from cell-agarose constructs due to the high levels of polysaccharides (55). From both samples, cDNA was generated from 500 ng–1 ug total RNA using a High Capacity Reverse Transcription Kit (4368814, Applied Biosystems, Waltham, MA), lysate was processed using the RNeasy Mini Kit (74104, Qiagen, Germantown, MD), and target genes were amplified using SYBR^®^ Select Master Mix (4385610, Applied Biosystems, Waltham, MA) in a Bio-Rad iQ5 (Bio-Rad, Des Plaines, IL). All primer sequences are listed in Supplementary Table S1. Gene expression changes were calculated by comparative threshold cycle method with data standardized to a sample control and normalized to GAPDH using the *ΔΔ*Cq method. Four biological replicates were used for each experimental group. Full descriptive statistics are located in the Supplementary Data. Negative controls substituting molecular grade water for cDNA were carried out for each primer for standard quality control.

### Total RNA-seq with GO and KEGG enrichment analyses

RNA-seq was performed by LC Sciences from RNA isolated from primary cells collected from 14-day-old wild type and NG2/CSPG4 knockout mice at passage four. RNA was isolated using the Qiagen RNeasy Mini Kit (79216 Qiagen, Germantown, MD). In short, a Poly(A) RNA sequencing library was prepared following Illumina’s TruSeq-stranded-mRNA sample preparation protocol. RNA integrity was checked with Agilent Technologies 2100 Bioanalyzer. Poly(A) tail-containing mRNAs were purified using oligo-(dT) magnetic beads with two rounds of purification. After purification, poly(A) RNA was fragmented using divalent cation buffer in elevated temperature. The DNA library construction is shown in the following workflow. Quality control analysis and quantification of the sequencing library were performed using Agilent Technologies 2100 Bioanalyzer High Sensitivity DNA Chip. Paired-ended sequencing was performed on Illumina’s NovaSeq 6000 sequencing system. For bioinformatics analysis, in house scripts were used to remove the reads that contained adaptor contamination, low quality bases and undetermined bases (56). Then sequence quality was verified using FastQC (http://www.bioinformatics.babraham.ac.uk/projects/fastqc/). HISAT2 (57) was used to map reads to the genome of ftp://ftp.ensembl.org/pub/release-101/fasta/mus_musculus/dna/. The mapped reads of each sample were assembled using StringTie (58). Then, all transcriptomes were merged to reconstruct a comprehensive transcriptome using perl scripts and gffcompare. After the final transcriptome was generated, StringTie (58) and edgeR (59) was used to estimate the expression levels of all transcripts. StringTie (58) was used to perform expression level for mRNAs by calculating FPKM. The differentially expressed mRNAs were selected with log2 (fold change) > 1 or log2 (fold change) < −1 and with statistical significance (*p* value < 0.05) by R package edgeR (59). Four biological replicates were used for the analysis.

### Western blot analysis

For *in vitro* and *in vivo* protein isolation of cultured cells, plates were washed with 1× PBS, lysed using an extraction reagent (M-PER, 78501, Thermo Fisher, Waltham, MA) with protease (cOmplete, 4693116001, Sigma, St. Louis, MO) and phosphatase (PhosSTOP, 4906845001, Sigma, St. Louis, MO) inhibitors. For *in vivo* tissues, tissue was homogenized in the lysis buffer. For *in vitro* protein isolation of the cell-agarose scaffolds, samples were rinsed in 1× PBS for 20 min, placed in Laemmli Buffer, boiled for 10 min, cooled on ice, and spun down for 2 h using a mini-spin column (Pierce Spin Cups, 69700, Thermo Fisher, Waltham, MA). For all samples, lysate insolubles were removed by centrifugation at 14,000*g* for 15 min at 4 °C. For monolayer cell and tissue samples tested for NG2/CSPG4, supernatant was incubated with Chondroitinase ABC (100330-1, AMSBio, Cambridge, MA) added at 0.05 units/ml for 2 h at 37 °C. For all samples, lysates were adjusted to a 1× Protein Sample Loading Buffer (928-40004, Licor, Lincoln, NE), heated at 99 °C for 10 min, separated on a 4%–15% sodium dodecyl sulfate polyacrylamide gel (SDS-PAGE), and analyzed by western blot with antibodies against NG2/CSPG4 (1 : 500, AB5320, Sigma-Millipore, Santa Cruz, CA), ERK (1 : 500, 9101S, Cell Signaling Technology, Danvers, MA), ERK phosphorylation at Thr202/Tyr204 and Thr185/Tyr187 (1 : 500, 4695T, Cell Signaling Technology, Danvers, MA), MEK phosphorylation at Thr202/Tyr204 (1 : 500, 4370T, Cell Signaling Technology, Danvers, MA), cRaf phosphorylation at ser338 (1 : 500, 56A6, Cell Signaling Technology, Danvers, MA), p90RSK phosphorylation at ser380 (1 : 500, D3H11, Cell Signaling Technology, Danvers, MA), and a β-actin mouse monoclonal control (1 : 1000, 926-42210, Licor, Lincoln, NE). Blots were imaged using a LI-COR Fluorescence Quantitative western blot. Fluorescence values were normalized to β-actin and standardized to experimental control samples. Four biological replicates were used for all western blots.

### Statistics

Statistical significance was assessed by one-way ANOVA. For multi-group comparisons, *post hoc* Bonferroni test were carried out. (SPSS, Chicago, IL). A statistically significant difference was considered as *p* < 0.05.

## Supplementary Material

Supplemental Material

## Data Availability

RNAseq data deposited in the Gene Expression Omnibus: Series GSE214077 https://www.ncbi.nlm.nih.gov/geo/query/acc.cgi?acc=GSE214077.

## References

[1] Tanaka E, Detamore M, Mercuri L. Degenerative disorders of the temporomandibular joint: etiology, diagnosis, and treatment. J Dent Res. (2008) 87(4):296–307. doi: 10.1177/154405910808700406 18362309

[2] Zhao Z, Li Y, Wang M, Zhao S, Zhao Z, Fang J. Mechanotransduction pathways in the regulation of cartilage chondrocyte homoeostasis. J Cell Mol Med. (2020) 24(10):5408–19. doi: 10.1111/jcmm.15204 32237113 PMC7214151

[3] Chu WC, Zhang S, Sng TJ, Ong YJ, Tan W-L, Ang VY, Distribution of pericellular matrix molecules in the temporomandibular joint and their chondroprotective effects against inflammation. Int J Oral Sci. (2017) 9 (1):43–52. doi: 10.1038/ijos.2016.57 28282029 PMC5379161

[4] Zelenski NA, Leddy HA, Sanchez-Adams J, Zhang J, Bonaldo P, Liedtke W, Type VI collagen regulates pericellular matrix properties, chondrocyte swelling, and mechanotransduction in mouse articular cartilage. Arthritis Rheumatol. (2015) 67(5):1286–94. doi: 10.1002/art.39034 25604429 PMC4414817

[5] Reed DA, Yotsuya M, Gubareva P, Toth PT, Bertagna A. Two-photon fluorescence and second harmonic generation characterization of extracellular matrix remodeling in post-injury murine temporomandibular joint osteoarthritis. PLoS One. (2019) 14(3):e0214072. doi: 10.1371/journal.pone.0214072 30897138 PMC6428409

[6] Polur I, Lee PL, Servais JM, Xu L, Li Y. Role of HTRA1, a serine protease, in the progression of articular cartilage degeneration. Histol Histopathol. (2010) 25 (5):599. doi: 10.14670/HH-25.599 20238298 PMC2894561

[7] Xu L, Servais J, Polur I, Kim D, Lee PL, Chung K, Attenuation of osteoarthritis progression by reduction of discoidin domain receptor 2 in mice. Arthritis Rheum. (2010) 62(9):2736–44. doi: 10.1002/art.27582 20518074 PMC2946478

[8] Burg MA, Tillet E, Timpl R, Stallcup WB. Binding of the NG2 proteoglycan to type VI collagen and other extracellular matrix molecules. J Biol Chem. (1996) 271 (42):26110–6. doi: 10.1074/jbc.271.42.26110 8824254

[9] Tillet E, Gential B, Garrone R, Stallcup WB. NG2 Proteoglycan mediates β1 integrin-independent cell adhesion and spreading on collagen VI. J Cell Biochem. (2002) 86(4):726–36. doi: 10.1002/jcb.10268 12210739

[10] Joo NE, Miao D, Bermúdez M, Stallcup WB, Kapila YL. Shedding of NG2 by MMP-13 attenuates anoikis. DNA Cell Biol. (2014) 33(12):854–62. doi: 10.1089/dna.2014.2399 25166220 PMC4248241

[11] Nishihara T, Remacle AG, Angert M, Shubayev I, Shiryaev SA, Liu H, Matrix metalloproteinase-14 both sheds cell surface neuronal glial antigen 2 (NG2) proteoglycan on macrophages and governs the response to peripheral nerve injury. J Biol Chem. (2015) 290(6):3693–707. doi: 10.1074/jbc.M114.603431 25488667 PMC4319034

[12] Larsen PH, Wells JE, Stallcup WB, Opdenakker G, Yong VW. Matrix metalloproteinase-9 facilitates remyelination in part by processing the inhibitory NG2 proteoglycan. J Neurosci. (2003) 23(35):11127–35. doi: 10.1523/JNEUROSCI.23-35-11127.2003 14657171 PMC6741053

[13] Asher RA, Morgenstern DA, Properzi F, Nishiyama A, Levine JM, Fawcett JW. Two separate metalloproteinase activities are responsible for the shedding and processing of the NG2 proteoglycan in vitro. Mol Cell Neurosci. (2005) 29 (1):82–96. doi: 10.1016/j.mcn.2005.02.001 15866049

[14] Nishiyama A, Lin X-H, Stallcup W. Generation of truncated forms of the NG2 proteoglycan by cell surface proteolysis. Mol Biol Cell. (1995) 6 (12):1819–32. doi: 10.1091/mbc.6.12.1819 8590808 PMC301335

[15] Reed DA, Zhao Y, Han M, Mercuri LG, Miloro M. Mechanical loading disrupts focal adhesion kinase activation in mandibular fibrochondrocytes during murine TMJ osteoarthritis. J Oral Maxillofac Surg. (2021) 79 (10):2058.e1–2058.e15. doi: 10.1016/j.joms.2021.05.001 PMC8500914 34153254

[16] Xu L, Polur I, Lim C, Servais J, Dobeck J, Li Y, Early-onset osteoarthritis of mouse temporomandibular joint induced by partial discectomy. Osteoarthr Cartil. (2009) 17(7):917–22. doi: 10.1016/j.joca.2009.01.002 PMC2941347 19230720

[17] Nayak T, Trotter J, Sakry D. The intracellular cleavage product of the NG2 proteoglycan modulates translation and cell-cycle kinetics via effects on mTORC1/FMRP signaling. Front Cell Neurosci. (2018) 12:231. doi: 10.3389/fncel.2018.00231 30131676 PMC6090502

[18] Ampofo E, Schmitt BM, Menger MD, Laschke MW. The regulatory mechanisms of NG2/CSPG4 expression. Cell Mol Biol Lett. (2017) 22(1):4. doi: 10.1186/s11658-017-0035-3 28536635 PMC5415841

[19] Tamburini E, Dallatomasina A, Quartararo J, Cortelazzi B, Mangieri D, Lazzaretti M, Structural deciphering of the NG2/CSPG4 proteoglycan multifunctionality. FASEB J. (2018) 33(3):3112–28. doi: 10.1096/fj.201801670R 30550356

[20] Yotsuya M, Bertagna AE, Hasan N, Bicknell S, Sato T, Reed DA. Neuron/glial antigen 2-type VI collagen interactions during murine temporomandibular joint osteoarthritis. Sci Rep. (2019) 9(1):56. doi: 10.1038/s41598-018-37028-1 30635602 PMC6329769

[21] Feutlinske F, Browarski M, Ku M-C, Trnka P, Waiczies S, Niendorf T, Stonin1 mediates endocytosis of the proteoglycan NG2 and regulates focal adhesion dynamics and cell motility. Nat Commun. (2015) 6:8535. doi: 10.1038/ncomms9535 26437238 PMC4600748

[22] Midwood KS, Salter DM. NG2/HMPG Modulation of human articular chondrocyte adhesion to type VI collagen is lost in osteoarthritis. J Pathol. (2001) 195(5):631–5. doi: 10.1002/path.985 11745701

[23] Jamil NS, Azfer A, Worrell H, Salter DM. Functional roles of CSPG4/NG2 in chondrosarcoma. Int J Exp Pathol. (2016) 97(2):178–86. doi: 10.1111/iep.12189 27292772 PMC4926050

[24] Makagiansar IT, Williams S, Mustelin T, Stallcup WB. Differential phosphorylation of NG2 proteoglycan by ERK and PKCα helps balance cell proliferation and migration. J Cell Biol. (2007) 178(1):155–65. doi: 10.1083/jcb.200612084 17591920 PMC2064431

[25] Barritt DS, Pearn MT, Zisch AH, Lee SS, Javier RT, Pasquale EB, The multi-PDZ domain protein MUPP1 is a cytoplasmic ligand for the membrane-spanning proteoglycan NG2. J Cell Biochem. (2000) 79(2):213–24. doi: 10.1002/1097-4644(20001101)79:2<213::AID-JCB50>3.0.CO;2-G 10967549 PMC3501957

[26] Ma D, Kou X, Jin J, Xu T, Wu M, Deng L, Hydrostatic compress force enhances the viability and decreases the apoptosis of condylar chondrocytes through integrin-FAK-ERK/PI3K pathway. Int J Mol Sci. (2016) 17(11):1847. doi: 10.3390/ijms17111847 27827993 PMC5133847

[27] Ryan JA, Eisner EA, DuRaine G, You Z, Hari Reddi A. Mechanical compression of articular cartilage induces chondrocyte proliferation and inhibits proteoglycan synthesis by activation of the ERK pathway: implications for tissue engineering and regenerative medicine. J Tissue Eng Regen Med. (2009) 3 (2):107–16. doi: 10.1002/term.146 19177463 PMC3777713

[28] Yang K, Wu Y, Cheng P, Zhang J, Yang C, Pi B, YAP And ERK mediated mechanical strain-induced cell cycle progression through RhoA and cytoskeletal dynamics in rat growth plate chondrocytes. J Orthop Res. (2016) 34 (7):1121–9. doi: 10.1002/jor.23138 26694636

[29] Fitzgerald JB, Jin M, Chai DH, Siparsky P, Fanning P, Grodzinsky AJ. Shear- and compression-induced chondrocyte transcription requires MAPK activation in cartilage explants. J Biol Chem. (2008) 283(11):6735–43. doi: 10.1074/jbc.M708670200 18086670

[30] Lu N, Malemud CJ. Extracellular signal-regulated kinase: a regulator of cell growth, inflammation, chondrocyte and bone cell receptor-mediated gene expression. Int J Mol Sci. (2019) 20(15):3792. doi: 10.3390/ijms20153792 31382554 PMC6696446

[31] Sun L, Zhao J, Wang H, Pan Y, Wang L, Zhang W-B. Mechanical stress promotes matrix synthesis of mandibular condylar cartilage via the RKIP-ERK pathway. J Mol Histol. (2017) 48(5–6):437–46. doi: 10.1007/s10735-017-9741-4 29119279

[32] Ding L, Heying E, Nicholson N, Stroud NJ, Homandberg GA, Buckwalter J, Mechanical impact induces cartilage degradation via mitogen activated protein kinases. Osteoarthr Cartil. (2010) 18(11):1509–17. doi: 10.1016/j.joca.2010.08.014 PMC3013628 20813194

[33] Vincent TL, Hermansson MA, Hansen UN, Amis AA, Saklatvala J. Basic fibroblast growth factor mediates transduction of mechanical signals when articular cartilage is loaded. Arthritis Rheumatol.. (2004) 50(2):526–33. doi: 10.1002/art.20047 14872495

[34] Goretzki L, Burg MA, Grako KA, Stallcup WB. High-affinity binding of basic fibroblast growth factor and platelet-derived growth factor-AA to the core protein of the NG2 proteoglycan. J Biol Chem. (1999) 274(24):16831–7. doi: 10.1074/jbc.274.24.16831 10358027

[35] Prasadam I, Mao X, Wang Y, Shi W, Crawford R, Xiao Y. Inhibition of p38 pathway leads to OA-like changes in a rat animal model. Rheumatology. (2012) 51 (5):813–23. doi: 10.1093/rheumatology/ker360 22240502

[36] Matsushita T, Chan YY, Kawanami A, Balmes G, Landreth GE, Murakami S. Extracellular signal-regulated kinase 1 (ERK1) and ERK2 play essential roles in osteoblast differentiation and in supporting osteoclastogenesis. Mol Cell Biol. (2009) 29(21):5843–57. doi: 10.1128/MCB.01549-08 19737917 PMC2772724

[37] Murakami S, Balmes G, McKinney S, Zhang Z, Givol D, de Crombrugghe B. Constitutive activation of MEK1 in chondrocytes causes Stat1-independent achondroplasia-like dwarfism and rescues the Fgfr3-deficient mouse phenotype. Genes Dev. (2004) 18(3):290–305. doi: 10.1101/gad.1179104 14871928 PMC338282

[38] Chen Z, Yue SX, Zhou G, Greenfield EM, Murakami S. ERK1 And ERK2 regulate chondrocyte terminal differentiation during endochondral bone formation. J Bone Miner Res. (2015) 30(5):765–74. doi: 10.1002/jbmr.2409 25401279 PMC4487783

[39] Pelaez D, Arita N, Cheung HS. Extracellular signal-regulated kinase (ERK) dictates osteogenic and/or chondrogenic lineage commitment of mesenchymal stem cells under dynamic compression. Biochem Biophys Res Commun. (2012) 417(4):1286–91. doi: 10.1016/j.bbrc.2011.12.131 22240026

[40] Vincent T, McLean C, Full L, Peston D, Saklatvala J. FGF-2 is bound to perlecan in the pericellular matrix of articular cartilage, where it acts as a chondrocyte mechanotransducer. Osteoarthr Cartil. (2007) 15(7):752–63. doi: 10.1016/j.joca.2007.01.021 17368052

[41] Yao Z, Chen P, Wang S, Deng G, Hu Y, Lin Q, Reduced PDGF-AA in subchondral bone leads to articular cartilage degeneration after strenuous running. J Cell Physiol. (2019) 234(10):17946–58. doi: 10.1002/jcp.28427 30834523

[42] Ghilardi SJ, O’Reilly BM, Sgro AE. Intracellular signaling dynamics and their role in coordinating tissue repair. Wiley Interdiscip Rev Syst Biol Med. (2020) 12 (3):e1479. doi: 10.1002/wsbm.1479 32035001 PMC7187325

[43] Loeser RF, Erickson EA, Long DL. Mitogen-activated protein kinases as therapeutic targets in osteoarthritis. Curr Opin Rheumatol. (2008) 20(5):581. doi: 10.1097/BOR.0b013e3283090463 18698181 PMC2892710

[44] Rosenfeldt H, Grinnell F. Fibroblast quiescence and the disruption of ERK signaling in mechanically unloaded collagen matrices. J Biol Chem. (2000) 275 (5):3088–92. doi: 10.1074/jbc.275.5.3088 10652290

[45] Nishiyama A, Dahlin K, Stallcup WB. The expression of NG2 proteoglycan in the developing rat limb. Development. (1991) 111(4):933–44. doi: 10.1242/dev.111.4.933 1879362

[46] Fukushi J, Inatani M, Yamaguchi Y, Stallcup WB. Expression of NG2 proteoglycan during endochondral and intramembranous ossification. Dev Dyn. (2003) 228(1):143–8. doi: 10.1002/dvdy.10359 12950088

[47] Midwood KS, Salter DM. Expression of NG2/human melanoma proteoglycan in human adult articular chondrocytes. Osteoarthr Cartil. (1998) 6 (5):297–305. doi: 10.1053/joca.1998.0128 10197164

[48] Poli A, Wang J, Domingues O, Planagumà J, Yan T, Rygh CB, Targeting glioblastoma with NK cells and mAb against NG2/CSPG4 prolongs animal survival. Oncotarget. (2013) 4(9):1527. doi: 10.18632/oncotarget.1291 24127551 PMC3824525

[49] Nicolosi PA, Dallatomasina A, Perris R. Theranostic impact of NG2/CSPG4 proteoglycan in cancer. Theranostics. (2015) 5(5):530. doi: 10.7150/thno.10824 25767619 PMC4350014

[50] Bougault C, Paumier A, Aubert-Foucher E, Mallein-Gerin F. Investigating conversion of mechanical force into biochemical signaling in three-dimensional chondrocyte cultures. Nat Protoc. (2009) 4(6):928–38. doi: 10.1038/nprot.2009.63 19478808

[51] Gosset M, Berenbaum F, Thirion S, Jacques C. Primary culture and phenotyping of murine chondrocytes. Nat Protoc. (2008) 3(8):1253–60. doi: 10.1038/nprot.2008.95 18714293

[52] Yotsuya M, Iriarte-Diaz J, Reed DA. Temporomandibular joint hypofunction secondary to unilateral partial discectomy attenuates degeneration in murine mandibular condylar cartilage. Bull Tokyo Dent Coll. (2020) 61 (1):9–19. doi: 10.2209/tdcpublication.2019-0008 32101827 PMC8304519

[53] ImageJ RW. Health UNIo. Maryland, USA: Bethesda (2012).

[54] Adler J, Parmryd I. Quantifying colocalization by correlation: the Pearson correlation coefficient is superior to the mander’s overlap coefficient. Cytometry Part A. (2010) 77(8):733–42. doi: 10.1002/cyto.a.20896 20653013

[55] Ogura T, Tsuchiya A, Minas T, Mizuno S. Methods of high integrity RNA extraction from cell/agarose construct. BMC Res Notes. (2015) 8(1):644. doi: 10.1186/s13104-015-1627-5 26537242 PMC4632373

[56] Martin M Cutadapt removes adapter sequences from high-throughput sequencing reads. EMBnet J. (2011) 17(1):10–2. doi: 10.14806/ej.17.1.200

[57] Kim D, Langmead B, Salzberg SL. HISAT: a fast spliced aligner with low memory requirements. Nat Methods. (2015) 12(4):357–60. doi: 10.1038/nmeth.3317 25751142 PMC4655817

[58] Pertea M, Pertea GM, Antonescu CM, Chang T-C, Mendell JT, Salzberg SL. Stringtie enables improved reconstruction of a transcriptome from RNA-seq reads. Nat Biotechnol. (2015) 33(3):290–5. doi: 10.1038/nbt.3122 25690850 PMC4643835

[59] Robinson MD, McCarthy DJ, Smyth GK. Edger: a bioconductor package for differential expression analysis of digital gene expression data. Bioinformatics. (2010) 26(1):139–40. doi: 10.1093/bioinformatics/btp616 19910308 PMC2796818

